# A larval zebrafish model of cardiac physiological recovery following cardiac arrest and myocardial hypoxic damage

**DOI:** 10.1242/bio.060230

**Published:** 2024-09-12

**Authors:** Warren Burggren, Regina Abramova, Naim M. Bautista, Regina Fritsche Danielson, Ben Dubansky, Avi Gupta, Kenny Hansson, Neha Iyer, Pudur Jagadeeswaran, Karin Jennbacken, Katarina Rydén-Markinhutha, Vishal Patel, Revathi Raman, Hersh Trivedi, Karem Vazquez Roman, Steven Williams, Qing-Dong Wang

**Affiliations:** ^1^ Developmental Integrative Biology Research Group, Department of Biological Sciences, University of North Texas, Denton, TX 76205, USA; ^2^ SVP and head of Research and Early Development, Cardiovascular, Renal and Metabolism (CVRM), BioPharmaceuticals R&D, AstraZeneca, Gothenburg 431 50, Sweden; ^3^ Bioscience Cardiovascular, Research and Early Development, Cardiovascular, Renal and Metabolism (CVRM), BioPharmaceuticals R&D, AstraZeneca, Gothenburg 431 50, Sweden

**Keywords:** Zebrafish, Myocardial infarction, Hypoxia, Regeneration, Cardiac output, Recovery, Animal model

## Abstract

Contemporary cardiac injury models in zebrafish larvae include cryoinjury, laser ablation, pharmacological treatment and cardiac dysfunction mutations. Although effective in damaging cardiomyocytes, these models lack the important element of myocardial hypoxia, which induces critical molecular cascades within cardiac muscle. We have developed a novel, tractable, high throughput *in vivo* model of hypoxia-induced cardiac damage that can subsequently be used in screening cardioactive drugs and testing recovery therapies. Our potentially more realistic model for studying cardiac arrest and recovery involves larval zebrafish (*Danio rerio*) acutely exposed to severe hypoxia (PO_2_=5-7 mmHg). Such exposure induces loss of mobility quickly followed by cardiac arrest occurring within 120 min in 5 days post fertilization (dpf) and within 40 min at 10 dpf. Approximately 90% of 5 dpf larvae survive acute hypoxic exposure, but survival fell to 30% by 10 dpf. Upon return to air-saturated water, only a subset of larvae resumed heartbeat, occurring within 4 min (5 dpf) and 6-8 min (8-10 dpf). Heart rate, stroke volume and cardiac output in control larvae before hypoxic exposure were 188±5 bpm, 0.20±0.001 nL and 35.5±2.2 nL/min (*n*=35), respectively. After briefly falling to zero upon severe hypoxic exposure, heart rate returned to control values by 24 h of recovery. However, reflecting the severe cardiac damage induced by the hypoxic episode, stroke volume and cardiac output remained depressed by ∼50% from control values at 24 h of recovery, and full restoration of cardiac function ultimately required 72 h post-cardiac arrest. Immunohistological staining showed co-localization of Troponin C (identifying cardiomyocytes) and Capase-3 (identifying cellular apoptosis). As an alternative to models employing mechanical or pharmacological damage to the developing myocardium, the highly reproducible cardiac effects of acute hypoxia-induced cardiac arrest in the larval zebrafish represent an alternative, potentially more realistic model that mimics the cellular and molecular consequences of an infarction for studying cardiac tissue hypoxia injury and recovery of function.

## Teaser

**Summary:** Contemporary cardiac injury models in zebrafish often lack the important element of myocardial hypoxia. We introduce a larval zebrafish model in which cardiac damage is induced in by exposure to severe ambient hypoxia.

## INTRODUCTION

Investigators have placed considerable focus on identifying stem cells, pharmaceuticals and other therapeutic methods that may facilitate a return of heart function following myocardial infarction, if not actual cardiac arrest (Davidson et al., 2021; Szydlak, 2023; Talman and Kivela, 2018; Wang et al., 2023). In the past, such research was often based on evaluating initial *in vitro* pharmacological effects in cell cultures that, depending upon the efficacy of drugs being tested, were then translated directly to standard testing in mammal models (Hughes et al., 2011). In the last several years, alternative (and often more tractable) models for investigation of myocardial infarction and cardiomyocyte regeneration have emerged. The zebrafish (*Danio rerio*) has become an invaluable animal model in this regard. The attributes of the zebrafish are many, and have been extensively reviewed, for example Domínguez-Oliva et al., 2023; Hodgson et al., 2018; Jagadeeswaran et al., 2016; and Yoganantharjah and Gibert, 2017; the ease and inexpensive nature of its housing, high fecundity, rapid sexual maturity and transparency of the embryos and larvae. The cardiovascular physiology of the zebrafish is also translatable to human health. In addition to having similar heart rates, the zebrafish ECG has clear P, QRS and T waves, and a QT duration that indicates a comparable repolarization time and similar electrophysiology, and comparable ventricular pressure volume characteristics (Salehin et al., 2021). Consequently, zebrafish are increasingly involved in rapid screening for the efficacy of cardio-active drugs (Bowley et al., 2022; de Abreu et al., 2021; Kithcart and MacRae, 2017; Pott et al., 2020). Importantly, the zebrafish model allows relatively rapid testing of drugs, stressors and or procedures as an efficient, cost-effective intermediate step between *in vitro* cell culture and the expensive and time-consuming *in vivo* mammalian cardiovascular testing.

Several models of creating cardio-injury that resembles myocardial infarction have been developed for adult zebrafish and other animals (Narumanchi et al., 2021; Rolland and Jopling, 2023). These include cryoinjury (Chablais and Jazwinska, 2012; Marques et al., 2021; Yu et al., 2018), ventricular resection (Poss et al., 2002; Sheng et al., 2021), laser ablation (Matrone et al., 2013), genetic manipulation (Abdul-Wajid et al., 2018; Duca and Cao, 2021; Sun et al., 2021) and pharmacological ablation of cardiomyocytes (Takada et al., 2017). These models have been quite effective in evaluating the potential for enhanced regeneration of cardiac tissue. Noteworthy, however, is that ‘effectiveness’ of cardiac recovery/regeneration at the physiological level has been indirectly inferred largely based on the observation of cardiomyocyte proliferation, usually detected by immunofluorescence or similar observations of cell function- for review see Sheng et al., 2021. Mechanical, genetic or pharmacological injury in the larval zebrafish heart doubtlessly reduces blood flow through the ventricle and the rest of the body but such measurements – essentially the ultimate proof of physiological recovery – are lacking.

An additional critique of existing methods for inducing cardiac damage and studying the heart's recovery is that current methods for inducing cardiac injury may fail to simulate myocardial infarction in one crucial aspect. Myocardial infarcts from severe ischemia in humans results, of course, in accompanying hypoxia in cardiac tissue, which in turn creates many cellular and molecular cascades in cardiac tissue (Francis and Baynosa, 2017; Ulaganathan et al., 2023). Yet, in the many studies in adult zebrafish using mechanical, pharmacological or genetic cardiac disruption are performed under normoxic conditions (if oxygen levels are even mentioned) and authors typically do not measure, nor even speculate, as to whether the experimentally induced injury concurrently induces tissue hypoxia. Two exceptions are a study using breakdance mutants (bre), where interference with cardiac output in larval zebrafish induces elevated Hif-1α protein expression, suggestive of tissue hypoxia (Kopp et al., 2010), and where hypoxia was measured in the remaining cardiac tissue following ventricular amputation (Jopling et al., 2012). Generally, however, there is little to no direct evidence that cardiac injury models compromise cardiac tissue and its subsequent regeneration in a way that mimics human myocardial infarction and the accompanying myocardial hypoxia.

Another critique of current infarction models and their cardiac effects in the zebrafish model is that the success or failure of cardiac regeneration has been assessed primarily by immunohistological or molecular endpoints (Ceci et al., 2018; Ryan et al., 2020; Sallin et al., 2015), and fail to link their results to a tractable physiological phenotype. Yet, complex and instructive actual physiological measurements are quite feasible. The transparent body wall of larval zebrafish allows observation of the beating heart of unrestrained, unanesthetized larval zebrafish (Perrichon et al., 2017). From these observations, not only can heart rate be measured (Barrionuevo and Burggren, 1999; Martin et al., 2019), but also stroke volume and cardiac output can be quantified (Bagatto and Burggren, 2006; Perrichon et al., 2017). Yet, the potential for zebrafish larvae as a more relevant and tractable model for evaluating restoration following injury of the all-important variable of cardiac output has not been exploited. Actual measurements of cardiovascular and physiological variables in studies using larval or adult zebrafish for assessing cardiac regeneration have largely been limited to measurement of the electrocardiogram (Lin et al., 2020, 2018; Ling et al., 2022; Santoso et al., 2020), although some echocardiography has been used (Hein et al., 2015; Lin et al., 2020; O'Riordan et al., 2023; Wang et al., 2017, 2018). Despite these advances, detailed assessment of the extent to which the physiological endpoint of cardiac output is actually protected or restored following cardiac injury and treatment, as opposed to inferring function from cellular markers, remains largely enigmatic in both adult and larval zebrafish.

Against this backdrop, in this study we developed and validated an alternative model of induced cardiac tissue hypoxia and recovery in the larval zebrafish based specifically on assessment of cardiac physiological function. Larval zebrafish undergoing rapid development have large capacity for self-repair (Matrone et al., 2013), even exceeding that of the adult zebrafish (Erhardt and Wang, 2022; Geng et al., 2021; Gupta et al., 2021). Rapid regeneration is particularly desirable in studies that aim to examine how pharmaceutical treatment can alter the extent and rate of recovery following cardiac injury. Here, we report that severe, acute hypoxic exposure induces an easily created and reproducible cardiac arrest, survived by 30-80% of larval zebrafish, that impairs heart rate, stroke volume and cardiac output for 2 days following induction, after which cardiac function is restored to control levels. By measuring heart rate, stroke volume, and cardiac output following hypoxia-induced cardiac arrest, this model goes beyond cellular/molecular markers with its assessment as a proxy for supposed changes in heart performance, and thus can serve as a highly tractable model for evaluating effects of pharmaceuticals on restoration of actual cardiac function.

## RESULTS

### Hypoxia effects on mobility

**Fig. 1.**
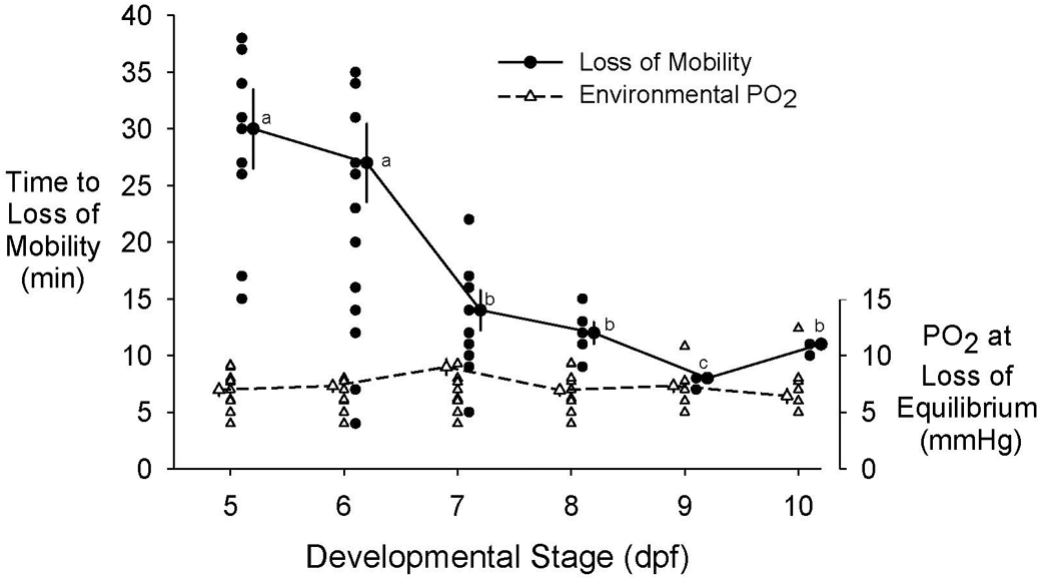
**Time to loss of mobility in the zebrafish (*Danio rerio)* as a function of developmental day (y-axis legend, on left) and water PO_2_ (y-axis legend, on right).** Control values (that is, loss of mobility non-hypoxia exposed larvae) are zero for each of the developmental days, and are not shown. For time to loss of equilibrium, significant differences (*P*<0.05) between means for each variable as development progresses from 5 to 10 days post fertilization, determined by one-way ANOVA followed by *post hoc* testing (Holm-Sidak), are indicated by different lower case letters, while non-significant differences are indicated by identical letters. There were no significant differences in mean values for environmental PO_2_s measured at time to loss of mobility (see Statistics section in Materials and Methods for additional details). Means±s.e.m. are plotted.

Hypoxic sensitivity was measured by time to loss of mobility following onset of ambient hypoxia. Time to LoM was ∼30 min at a PO_2_ of 5-7 mmHg at 5 dpf (Fig. 1). However, time to LoM at a PO_2_ of 5-7 mmHg decreased significantly (*P*<0.05, ANOVA) as larvae developed, falling to just 8-15 min for larval stages 7-10 dpf.

### Hypoxia effects on cardiac function

#### Cardiac arrest by hypoxia

**Fig. 2.**
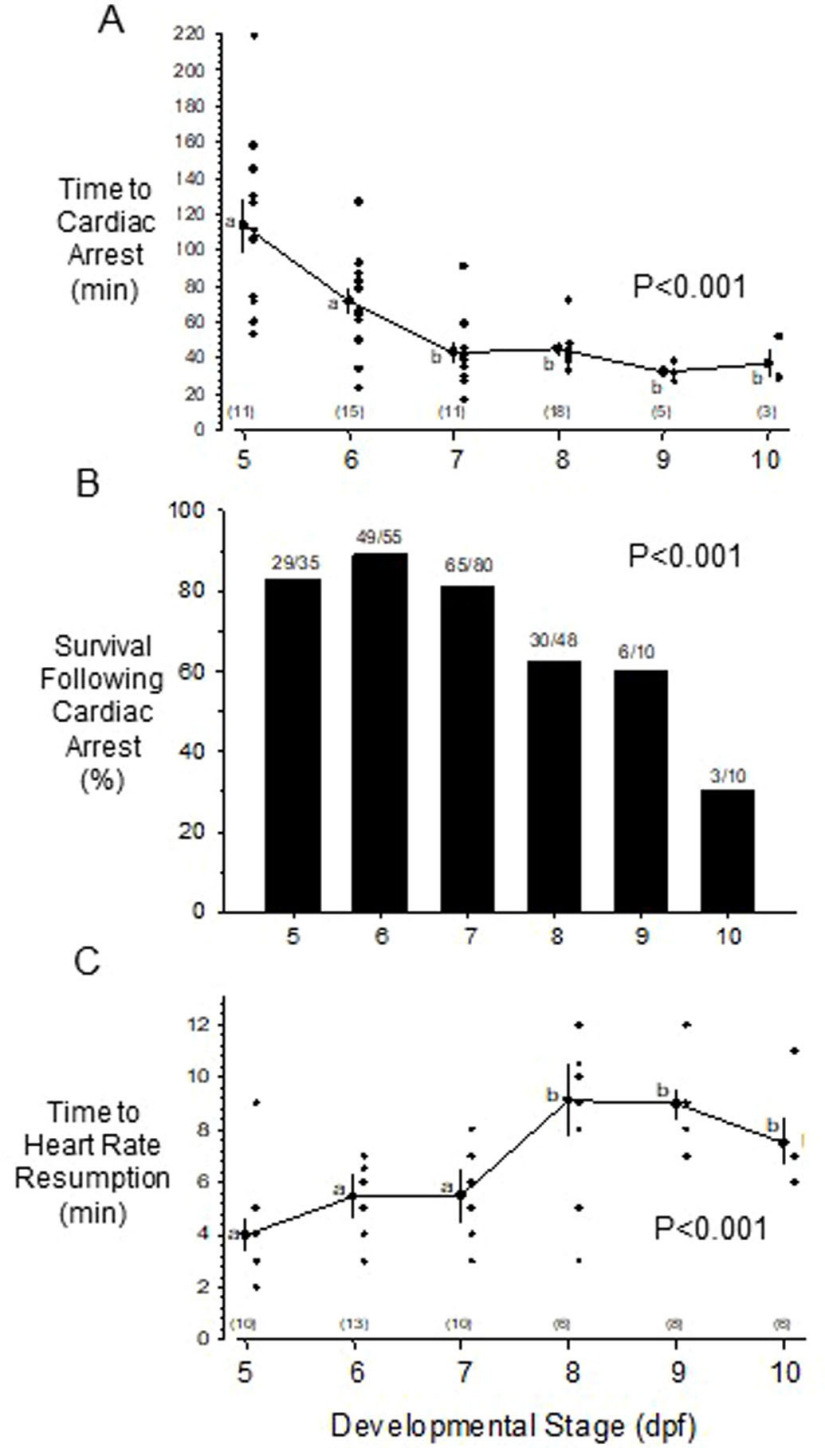
**Cardiac arrest and recovery following exposure to hypoxia (ambient PO_2_ of** ∼**-79 mmHg) as a function of development in the zebrafish.** (A) Time to cardiac arrest induced by hypoxia. (B) Survival following cardiac arrest. Numbers of surviving larvae versus total tested population are indicated above each bar. Significance of survival differences was determined with Fischer's Exact Test. (C) Time required for resumption of heartbeat following return to air saturated water in larvae surviving severe hypoxia. *P* values for developmental stage effects, determined by one-way ANOVAS for each variable, are indicated for each variable towards the right of the figure. Data for panels (A) and (C) were recorded to nearest minute, so not all individual points are apparent. Means±s.e.m. are plotted. Lower case letters in panels A and C indicate significant differences between developmental stages as determined by one-way ANOVAS (see Legend to Fig. 1 for additional details). See Statistics section in Materials and Methods for additional details. *N* values for each day are in parentheses at the bottom of the graphs (A and C) or above the bars (B).

All larvae tested showed cardiac arrest in severely hypoxic water (5-7 mmHg) given sufficient exposure time. Time to cardiac arrest differed significantly (*P*<0.001) between developmental stages (Fig. 2A). The youngest larvae tested (5 dpf) showed the greatest cardiac resistance to severe hypoxia, with an average time to cardiac arrest of 118±16 min. This value fell significantly to 75±7 min at 6 dpf. From 7-10 dpf, time to cardiac arrest was approximately the same in all stages, at about 30-45 min of hypoxic exposure.

Survival following hypoxia-induced cardiac arrest was highly dependent upon developmental stage (*P*<0.001) (Fig. 2B). Approximately 80-90% of the dpf 5-7 larvae suffering cardiac arrest recovered when returned to aerated water, as determined by resumption of heartbeat and return of body mobility. Beyond dpf 7, however, survival was less assured following hypoxic exposure, and had fallen to approximately 30% by dpf 10.

Recovery times from cardiac arrest – i.e. time required for heartbeat to resume following arrest in severely hypoxic water – are shown in Fig. 2C. Corresponding with the other indicators of hypoxia sensitivity (time to LoM, time to cardiac arrest, survival %), recovery was significantly more rapid (*P*<0.001, ANOVA) in the youngest larvae. Dpf 5-7 larvae typically required ∼4-6 min for the heart to resume beating following return to normoxic water, while larvae from 8-10 dpf typically required a significantly longer 6-9 min for resumption of heartbeat.

#### Post-cardiac arrest cardiac performance

**Fig. 3.**
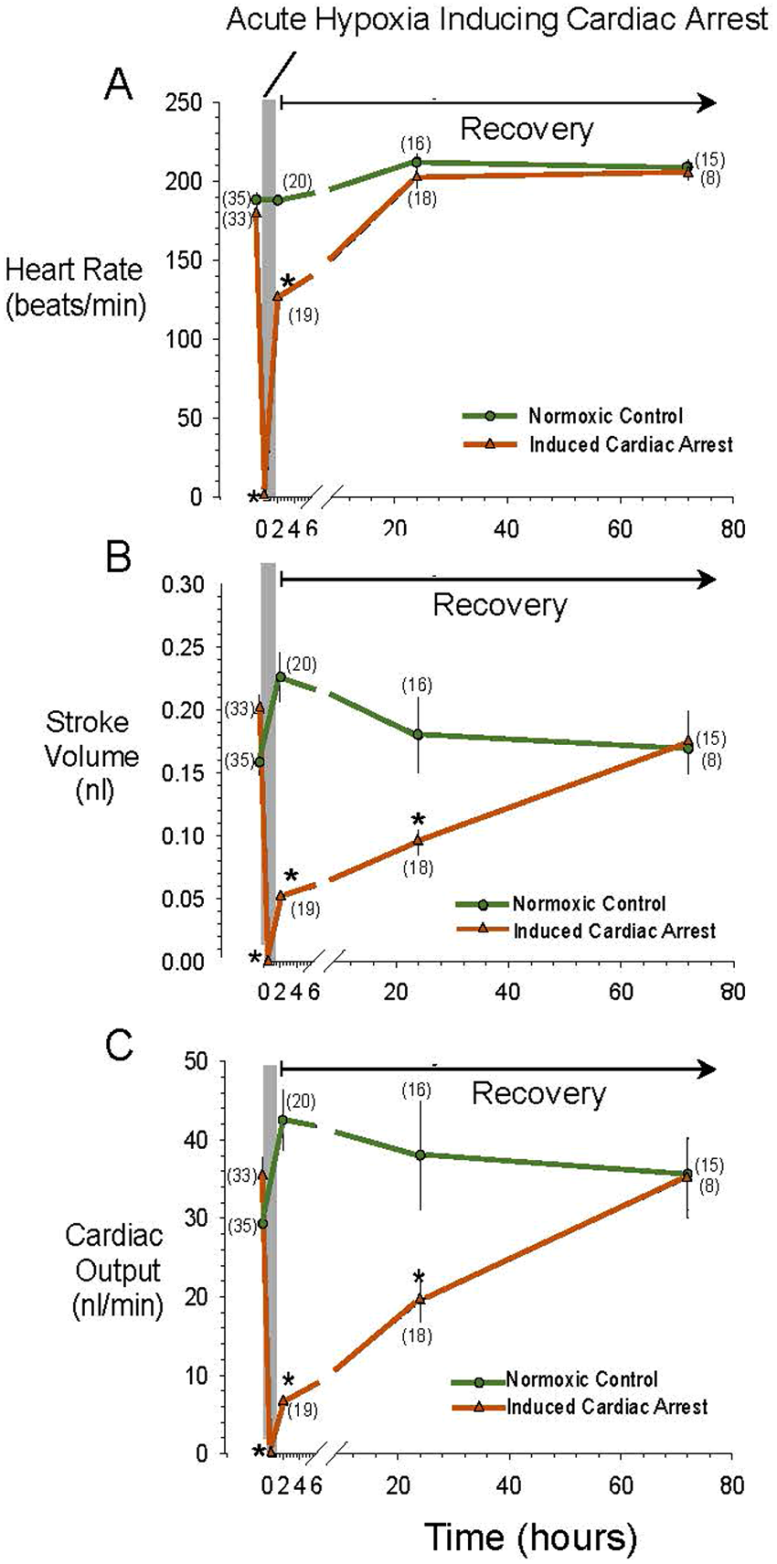
**Time course of changes in heart rate (A), stroke volume (B), and cardiac output (C) in 5-7 day larval zebrafish in control larvae and in larvae following induction of cardiac arrest by acute hypoxic exposure (PO_2_∼5-7 mmHg).** (See Materials and Methods for details of hypoxic exposure and treatment of controls). Cardiac values in the control population, exposed only to normoxia but otherwise treated identically to the hypoxia-exposed fish (green symbols and lines), as well as the responses to exposure to severe hypoxia of the experimental population (brown symbols and lines) were determined in individual larvae at each indicated time point. For all three cardiac variables in the experimental populations, induced cardiac arrest resulted in significant (*P*<0.001) changes, whereas no significant changes (*P*>0.4) occurred in the sham population only exposed to normoxia. Means±s.e.m. are presented. Error bars may be smaller than symbol size. Numbers in parentheses are *N* values for each observation. An * indicates a significant difference of the value for the experimental population from the control population at the *P*<0.001 level, determined by one-way ANOVA.

Fig. 3 illustrates cardiac performance in the control population, and in the experimental population before, during and 80 h after cardiac arrest at 5-7 dpf. In the experimental group experiencing cardiac arrest, each of the measured indicators of cardiac performance – heart rate, stroke volume and cardiac output – were significantly (*P*<0.001, two-way ANOVA) affected by hypoxia-induced cardiac arrest (Fig. 3).

Heart rate in the control population of 5-7 dpf larvae was ∼190 bpm for the duration of the 80 h measurement period and showed no significant differences (*P*>0.05) at any time point. Heart rates at t=0 of the control population in normoxia and in the experimental population prior to the start of hypoxic exposures were 188±5 bpm and 179±2 bpm, respectively. Following hypoxia-induced cardiac arrest and eventual resumption of heartbeat in the experimental population, heart rate was still only 126±4 bpm 2 h into recovery, a significant (*P*<0.001) decline of ∼30% from pre-exposure values and from values evident in the control populations at 2 h (Fig. 3A). However, heart rate in the experimental population recovered to values not significantly different (*P*>0.05) from pre-exposure values or control population values by ∼24 h and 72 h following cardiac arrest

Stroke volume in the control population was 0.17-2.3 nL for the duration of the 80 h measurement period, and showed no significant differences (*P*>0.05) at any time point. In the experimental population experiencing cardiac arrest, there was a severe and long-lasting decline in stroke volume following hypoxia-induced cardiac arrest. Stroke volume decreased significantly (*P*<0.001) by ∼75% from 0.200±0.001 nL in normoxia prior to hypoxia-induced cardiac arrest to just 0.051±0.005 nL ∼2 h after resumption of heartbeat (Fig. 3B). Although heart rate had resumed control values, stroke volume was still approximately 50% depressed at t=24 h, but recovered by t=72 h.

Cardiac output in the control population was 30-40 nL/min for the duration of the 80 h measurement period, and showed no significant differences (*P*>0.05) at any time point. In the experimental population, unsurprisingly given that cardiac output is the product of heart rate and stroke volume, cardiac output was sharply and significantly (*P*<0.001) reduced by 80% from a pre-arrest value of 35.5±2.2 nL/min to just 6.6±0.7 nL/min at t=2 h. This severe reduction in cardiac output was still evident at t=24 h, when heart rate was back at control values, but stroke volume was still significantly depressed by ∼50% compared to the pre-arrest values or those of the normoxia population. Full recovery of cardiac output to control values occurred by 72 h.

#### Immunohistochemical staining

**Fig. 4.**
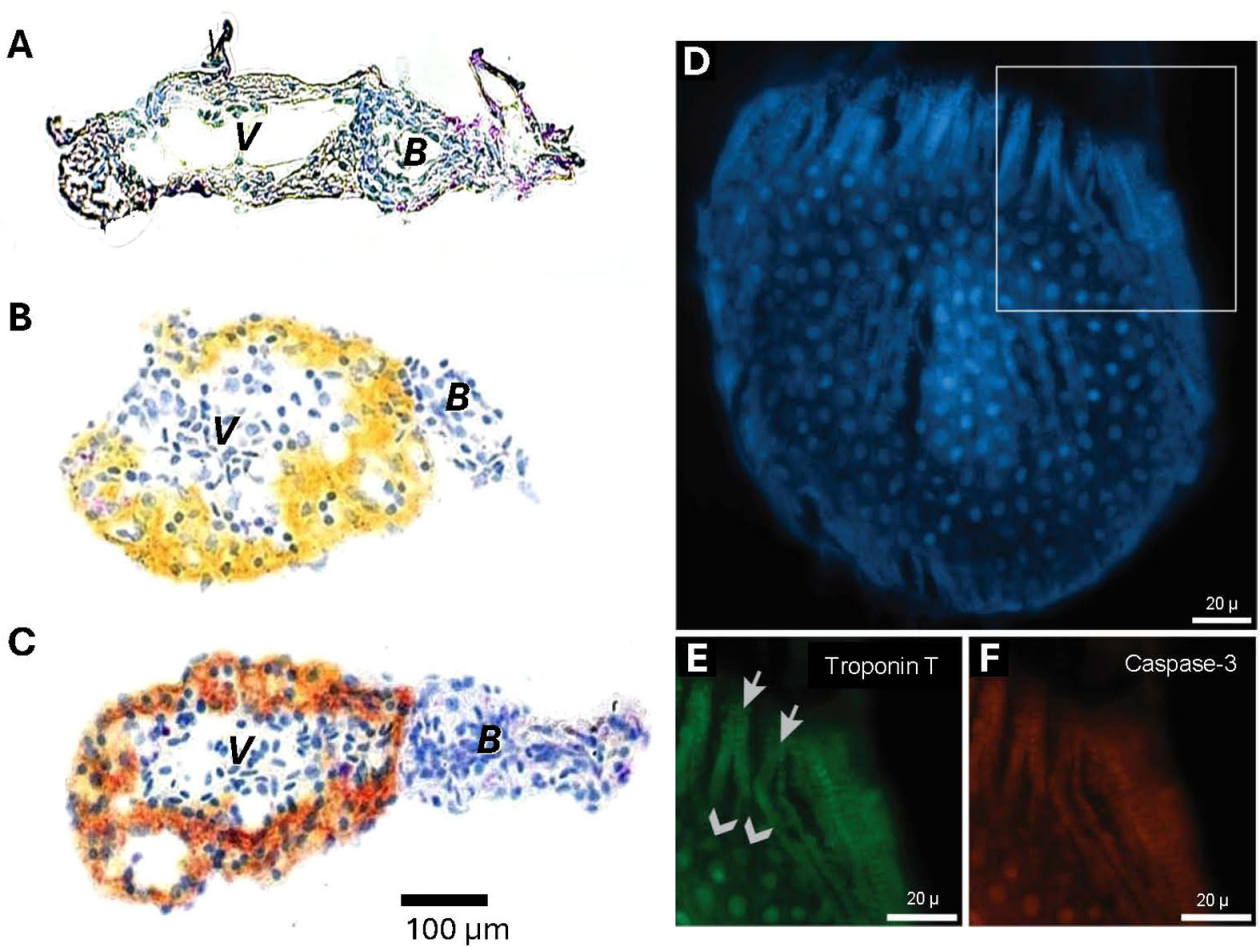
**Chromogenic duplex immunohistological staining of the 7 dpf larval zebrafish heart to show cardiomyocyte damage 2 h after cardiac arrest and resumption of heartbeat following severe, acute hypoxic exposure.** The ventricle (*V*) contains cardiac muscle cells, while the attached bulbus arteriosus (*B*) contains only smooth muscle cells. Panels A-C show representative photographs obtained by staining performed on the hearts of five 7 dpf larvae 2** **h following complete cardiac arrest and resumption of heartbeat. Staining was performed to reveal cardiac troponin T (cTnT) and cleaved caspase-3 (cC3), the latter a biomarker for apoptosis. Slides were counterstained with hematoxylin. (A) Hearts of control larvae (no hypoxic exposure) were negative for Caspase 3, showing only hematoxylin counterstained nuclei (blue). (B) Yellow staining in the ventricular myocardium indicates cTnT expression in cardiac muscle cells. (C) Deep orange/brown staining represents co-expression of cTnT and cC3 in damaged cardiac muscle cells. Note that in panels (B) and (C) there is a complete lack of cTnT or cC3 staining in the bulbus arteriosus, which contains only smooth muscle. Panels D-F show co-localization of cardiomyocytes and Caspase-3 to identify cardiomyocytes undergoing apoptosis. Staining was performed on three larvae 2 h following complete cardiac arrest and resumption of heartbeat in a 7** **dpf zebrafish larvae. (D) Image of background actin stain in blue depicts a section of ventricular tissue. (E) Magnified image from area of white outline in D shows both cardiomyocytes (arrows) and endocardial cells (chevrons) expressing Troponin T (green). (F) Co-localization of Caspase-3 expression (red) in the same cardiac cells depicted in panel E.

To demonstrate that cardiomyocytes themselves were being damaged by hypoxic exposure and cardiac arrest, ventricles from larvae experiencing cardiac arrest were subjected to qualitative immunohistochemical staining. Fig. 4A shows hematoxylin staining of nuclei of both of myocardiocytes and red blood cells within the lumen of the ventricle and bulbus arteriosus of a control 7 dpf larvae not experiencing hypoxia-induced cardiac arrest. Fig. 4B and C is from heart tissue sampled 2 h after resumption of heart beat following cardiac arrest, showing evidence of cardiomyocyte damage in the form of staining for cardiac troponin T (cTnT), a biomarker of cardiomyocyte damage (Gerhardt and Ljungdahl, 1998) and Caspase-3 (C3), a biomarker for apoptosis (Porter and Jänicke, 1999). Importantly, the bulbus arteriosus containing only smooth muscle showed no staining for these biomarkers. To ensure that cardiomyocytes, rather than other cell types showing damage following cardiac arrest, Fig. 4D-F show tissue sampled 2 h after resumption of heartbeat following cardiac arrest. Co-localization of troponin T and Caspase-3 was apparent in cardiomyocytes, as well as in endocardial cells.

## DISCUSSION

### Larval zebrafish cardiovascular measurements under control conditions

Heart rate, stroke volume and cardiac output have been measured in zebrafish larvae and other fishes at similar developmental stages and temperatures in numerous studies (Khursigara et al., 2017; Magnuson et al., 2020; Pasparakis et al., 2019; Perrichon et al., 2017). The cardiac values from the present study measured in control conditions (normoxia) on 5-7 dpf zebrafish larvae are in close agreement with those studies highlighting the reliability and reproducibility of this system for assessing cardiac function.

### Larval zebrafish cardiovascular measurements and hypoxia sensitivity

Hypoxia effects in larval zebrafish have involved studies of ventilation (Abdallah et al., 2015; Aukland, 2001; Coe et al., 2017; Mandic et al., 2019; Perry et al., 2023) and metabolism (Barrionuevo and Burggren, 1999; Ho and Burggren, 2012; Mandic et al., 2020; Napolitano et al., 2019; Pan and Hunt von Herbing, 2017). While changes in larval cardiac performance under conditions of mild to moderate hypoxia have been investigated (Barrionuevo and Burggren, 1999; Barrionuevo et al., 2010; Cypher et al., 2017; Schwerte, 2009; Tzaneva and Perry, 2016), knowledge of resistance of the heart to severe hypoxia in early larval stages, and how that changes during development, is fragmentary. In one of the few such studies, examining the clinical problem of reperfusion injury, 2 dpf zebrafish larvae were subjected to 48 h of hypoxia followed by 2-5 h of reoxygenation to simulate reperfusion injury following simulated myocardial ischemia (Zou et al., 2019). Cardiac function declined at the end of this period of hypoxia and was still impaired 72 h later.

Our study has revealed that cardiac resistance to severe hypoxia, measured by time to heartbeat cessation, time required for recovery, and whole animal survival, is highest in early larval stages, but decreases as development progresses from 5 dpf to 10 dpf, especially in the later days of this developmental range. Larval zebrafish from 5-10 dpf show considerable developmental changes associated with both the morphology and physiology of the cardiovascular system (Burggren and Bagatto, 2008; Burggren et al., 2017). Therefore, increasing cardiovascular susceptibility to hypoxia as development continues may be reflected in these changes.

The mechanisms underlying hypoxic resistance, and how and why it wanes during development, are not entirely clear. Changes in mRNA expression of *tnnt2* (troponin T), *bnp* (B-type natriuretic peptide) and *hif1α* (multiple hypoxia-related effects) result from simulated reperfusion in zebrafish larvae (Zou et al., 2019). Cardiac ‘stunning’ occurring after a temporary post-ischemic cardiac event manifests as mechanical dysfunction and involves production of catecholamines and endothelin, myocardial inflammation, and coronary occlusion (Guaricci et al., 2018; Kloner, 2020; Pomblum et al., 2010), and occurs in a number of animals (Weisel, 1993). The observed temporary disruption to cardiac performance following cardiac arrest in larval zebrafish likely represents actual cell death rather than just cardiac stunning, based on our staining with an apoptotic marker. However, additional immunohistological and other molecular examination will be required to quantify apoptosis, etc.

### Larval zebrafish as a cardiac ischemia model

Our goal was the development of a more comprehensive model for cardiac tissue hypoxia as might be associated with, for example, myocardial infarction. We focused on 5-7 dpf larval zebrafish for several reasons. First, prior to 5 dpf the zebrafish heart is undergoing looping and its chambers ballooning as organogenesis occurs. After 5 dpf, however, the zebrafish heart approaches its adult gross morphological configuration, organogenesis is largely complete, and the heart is primarily growing rather than forming *per se* (Burggren and Bagatto, 2008; Burggren et al., 2017; Kemmler et al., 2021; Martin and Waxman, 2021). Second, it was desirable to have the heart of hypoxia-exposed larvae be sufficiently damaged to show a severely impacted performance (especially in cardiac output) and actually result in the death of some larvae, yet also retain the capacity of survivors to recover from damage. Third, as a practical matter, beyond ∼7-8 dpf the increasing amounts of pigment in the body wall covering the heart renders measurements of stroke volume and cardiac output less reliable (Bagatto and Burggren, 2006; Perrichon et al., 2017). Moreover, examining zebrafish larvae in earlier development at stages, when they were resistant to hypoxia-induced cardiac arrest, allowed for higher survival rates: 85-90% at 5 dpf compared to 30% at 10 dpf (Fig. 2B). By using 5-7 dpf larvae in our study, severe hypoxia clearly impacted heart performance for at least 24 h. This decrease in heart performance may be correlated with immunohistochemical evidence of cardiomyocyte apoptosis at hour 2 (Fig. 4). Troponin T and Caspase-3 appear to colocalize in cardiomyocytes, as well as in endocardial cells (Fig. 4B, C), indicating that cellular damage is occurring from this protocol and that this experimental model will allow for monitoring of observed deterioration in physiological performance in future comparisons.

Larvae surviving cardiac arrest and damage naturally achieved restoration of cardiac function in the post exposure period, with full functionality restored by 72 h following cardiac arrest (Fig. 3). The heart of the zebrafish adult shows remarkable regenerative capabilities (compared to adult mammals) following damage (Botos et al., 2023; Cahill et al., 2017; Poss et al., 2002; Ross Stewart et al., 2022). Regenerative capabilities are likely to be even greater in the larval zebrafish.

### Model potential for drug testing for cardiac proliferation and recovery

**Fig. 5.**
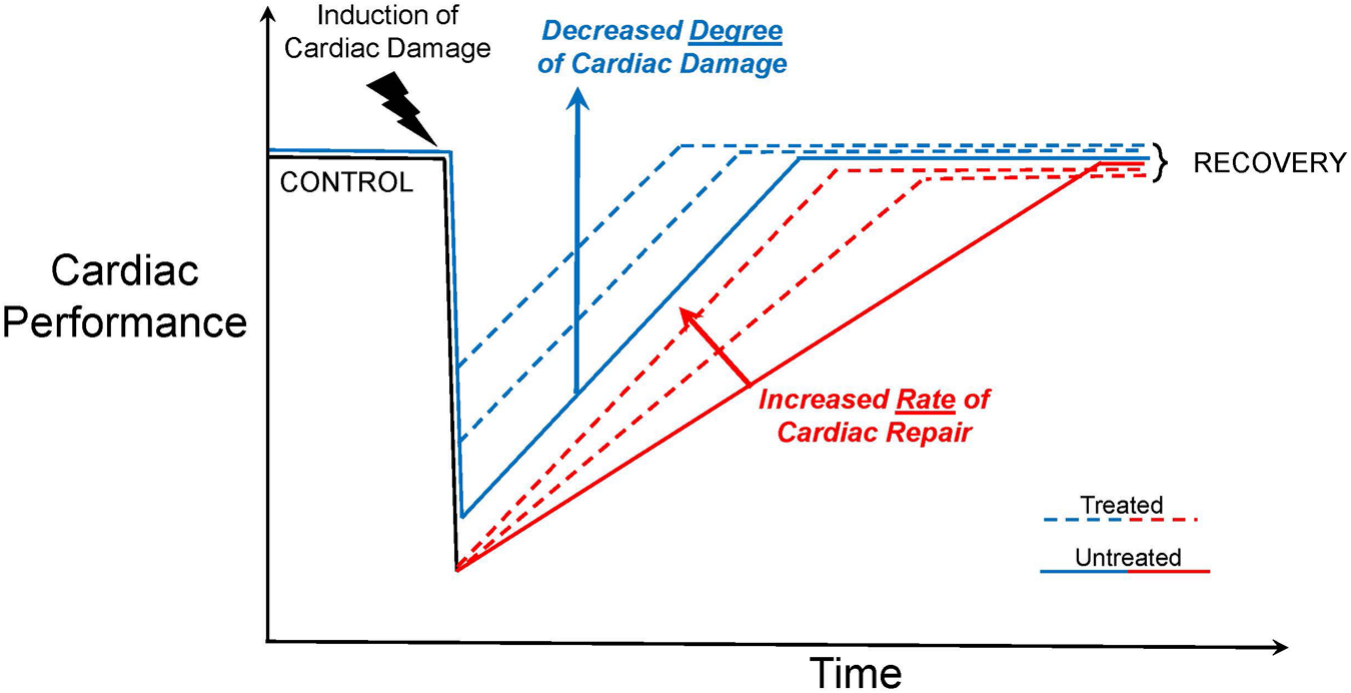
**Schematic of possible therapeutic outcomes following induction of hypoxic-induced cardiac arrest using the larval zebrafish model for myocardial infarction.** Several possible outcomes following a pharmacological or other intervention following cardiac arrest are illustrated. (A) Cardiac function is restored to control values by treatment. In one scenario (blue dashed lines, the *extent* of damage is reduced, potentially showing a dose-dependent response. In the second scenario (red dashed lines), the rate of cardiac repair is enhanced, again showing potential dose-dependency. Also possible is that both the degree of damage is reduced, *and* the rate of repair is increased. (B) Cardiac function is not only restored to control values by treatment but is elevated above them. In this scenario there is an ‘overshoot’ whereby drug treatment stimulates cardiac function to levels higher than would be expected for control.

Cardiomyocyte regeneration and restoration of cardiac output using the larval zebrafish hypoxia-induced cardiac arrest model is likely to be influenced by changes in both the magnitude of the original disruption and the recovery time to control values (potentially in a dose-response fashion) (Fig. 5). Thus, compared with the untreated trajectory for diminished and then restored cardiac performance induced by cardiac arrest, there could be a variably diminished effect (Fig. 5A). Alternatively, the rate of regeneration could be enhanced, with full recovery occurring sooner than in the untreated population (Fig. 5B**)**. Irrespective of the desired effect, the >24 h period of severely reduced cardiac output resulting from transient hypoxia-induced cardiac arrest should provide a sufficiently lengthy therapeutic window for assessment of pharmaceutical compounds that may enhance recovery from cardiac events. Indeed, at least in cell culture and microphysiological systems, modelled cardiac events have shown the potential for investigating mechanisms of recovery following cardiac events, such as cardiomyocyte remodeling (Ding et al., 2020; Jennbacken et al., 2019; Mills et al., 2019). Here, based collectively on available data, we suggest that the recovery trajectory of cardiovascular physiology following hypoxic treatment is a sensitive indicator of cardiovascular injury in the larval zebrafish.

### Advantages and limitations of the zebrafish larva cardiac arrest model

Several advantages exist for using the hypoxia-induced cardiac arrest zebrafish model for studying recovery from myocardial infarction. As already alluded to, zebrafish are inexpensive to house, can be prolific breeders, and are deeply understood in terms of their genome. Importantly, larvae do not need to be anesthetized for cardiovascular measurements, which can be made while the larva is partially immobilized, reducing stress beyond the hypoxic induction of cardiac arrest. Moreover, no surgical intervention, including opening of the body wall and associated trauma, is involved, unlike with cryoinjury or ventricle resection. Additionally, by controlling water chemistry, dosing regimens and the induction of cardiac damage by sudden, severe exposure to hypoxia allows for relatively rapid preparation of subjects for subsequent examination in larval zebrafish. Thus, this model retains the complexity of a whole organism, while offering some *in vitro* experimental characteristics and a higher throughput alternative to the mouse and a more realistic alternative than cell-based microphysiological systems such as organoids, organ on chip and 2D cell culture.

While the techniques used in the current study have examined individual larvae in sequence, videomicroscopy can be scaled up to observation of multiple larvae simultaneously (Martin et al., 2019). This approach for assessment of cardiovascular performance during the regeneration process can yield relatively rapid results. A final example of an advantage of the larval hypoxia-induced cardiac arrest model involves time scale. The recovery process is relatively rapid, with recovery of heart rate within 24 h and of stroke volume and cardiac output within 72 h (Fig. 3). This allows relatively rapid collection of results, given that some cardiac regeneration experiments in mice, for example, can take 2-6 weeks or longer (Shafiq et al., 2018; Virag and Murry, 2003).

The hypoxia-induced cardiac arrest model is not without some limitations, as all such models have. The ability to measure not only heart rate but also stroke volume (and thus calculate cardiac output) declines with advancing development because of the increase in pigmentation of the larval body wall. By approximately 8-10 dpf, the body wall has become sufficiently opaque as to prevent visualization of the beating heart. In theory, the observation period could be extended by using albino fish, which have been generated using CRISPR/Cas9 technology (Fang et al., 2018; Liu et al., 2018) or by use of pigment mutation strains such as *crystal* or various casper mutants (Antinucci and Hindges, 2016). The production of melanin could also be pharmacologically disrupted using phenothiourea (Guillot et al., 2016), although this compound is not without its serious side effects (Bohnsack et al., 2011; Li et al., 2012).

Adopters of the larval zebrafish model of hypoxic-induced cardiac arrest also should be aware that cardiac damage and recovery following severe hypoxia represents a potentially complex interaction between regeneration and development. That is, pharmacological or other interventions might be expected to (ideally) increase the *rate* of restoration that will naturally occur (Fig. 5). However, restoration of damaged heart tissues results from a combination of regeneration and natural processes of development - the latter absent in mature animals. As such, this developmental model offers a robust and sensitive indicator of cardiogenic effects, in a complex platform.

### Future directions

It is generally agreed that reduced brain ischemia is the proximate issue affecting ultimate survival, rather than diminished blood flow generally in the systemic circulation per se (Hornby et al., 2018). In this context, it would be very interesting to perform physiological, morphological and behavioral assays on surviving larval zebrafish suffering cardiac arrest to determine if there were any transient or lasting effects, possibly manifested through central neural effects. Certainly, a variety of fish behavioral assessments are in common use (Hamilton, 2018; Khursigara et al., 2023). As well, the effects of a second challenge to surviving larvae after full recovery would be very interesting to conduct to see if there is a protective effect induced following survival of an earlier cardiac arrest – and if so, determining the mechanism.

## METHODS AND MATERIALS

### Embryo acquisition and larval maintenance

Adult (8-18 month old) *AB* strain zebrafish (*Danio rerio*) from the breeding stock at the University of North Texas were acclimated for 2 weeks under recommended husbandry conditions for this species (Westerfield, 2007). For breeding, female and male fish were transferred to separate 10 L tanks. At the start of the light period the following morning, four males and four females were placed together for courtship and mating. The resulting eggs were collected, rinsed with deionized water and placed in clean conditioned aquarium water at 27±0.5°C.

Larvae were randomly selected from the hatching eggs (2 days post fertilization, dpf) and were maintained in 1 L tanks. At 5 dpf, larvae were transferred to a new container and fed with live paramecia twice daily. From 8 dpf, they were fed with both paramecia and live brine shrimp. Larvae AB zebrafish develop as female-like fish until the process of sexual differentiation takes place at around 20-35 dpf (Bautista et al., 2023). Thus, the larvae used throughout this study (5-10 dpf) cannot be sexed, so it is assumed that equal numbers of males and females were assessed.

At the completion of measurements, larvae were euthanized by immersion for 10 min in 1:500 MS-222 buffered to 7.5 with NaHCO_3_ followed by placement in zinc-buffered formalin (Z-fix^®^, Anatech, Ltd.)

### Hypoxia effects on mobility and cardiac arrest characteristics

#### Observation arena

Experiments assessing how severe hypoxia affected loss of equilibrium, time to cardiac arrest, survival following cardiac arrest, and time for resumption of heart rate in surviving larvae were performed on larvae between 5 and 10 dpf. Severely hypoxic water (PO_2_ of 5-7 mmHg) was produced by bubbling nitrogen gas through a reservoir of aquarium water maintained at 28°C. This level of hypoxia was chosen because pilot tests indicated that a PO_2_ of 5-7 mmHg relatively quickly created loss of mobility (LoM) and cardiac arrest (see Results for quantitative effects). Larvae were placed in a glass dish containing this severely hypoxic water and located on a Leica S9D microscope fitted with a heated stage to maintain 28°C and a Leica MC190 HD camera. Once placed in the severely hypoxia water, larvae were timed until they exhibited spontaneous LoM and cessation of heartbeat (cardiac arrest). Cardiac arrest was confirmed by observing the movements (or lack thereof) of the single ventricle and atrium through the transparent body wall. Immediately following hypoxic exposure and timing and verification of cardiac arrest, larvae were gently transferred by pipette into individual wells in a six-well plate containing ∼2 ml of fully aerated water (PO_2_ ∼150 mmHg) at 28°C for recovery. Survival was assessed by whether the heart spontaneously resumed beating following hypoxia-induced cardiac arrest – no intervention was taken by the experimenters other than returning larvae to fully oxygenated water. In surviving larvae, time to spontaneous restoration of heartbeat was recorded in minutes from the moment of cardiac arrest to the moment when heart rate was resumed.

Following a 2 h recovery period in normoxic water, surviving larvae were gently transferred by pipette into a Petri dish filled with 3% methyl cellulose and normoxic aquarium water to a depth of 2 mm and maintained at 28±0.2°C. Methyl cellulose is a viscous substance that is commonly used to restrain larval fishes *in lieu* of anesthesia (Bautista et al., 2023; Perrichon et al., 2017). The Petri dish was again placed under the microscope for brief observation of cardiac activity of the larva, being careful not to jar or otherwise disturb the larva.

For comparison to the experimental population, a control (sham) population consisted of larvae treated exactly like the experimental population suffering induced cardiac arrest except the controls only experienced normoxia (PO_2_ 145-150 mmHg) produced by bubbling air into the media, rather than nitrogen. This control population was maintained normoxia, transferred to the observation Petri dish, and cardiac data recorded exactly as for the experimental nitrogen exposed population.

#### Measurement protocol for cardiac variables

Larvae were monitored for the cardiac variables of heart rate (beats per minute, bpm), stroke volume (nL), and cardiac output (nL/min) using video microscopy. These techniques, developed over the previous few decades for larval fishes, have been reviewed and assessed (Perrichon et al., 2017). Briefly, digital images of the beating heart in the intact larvae were recorded using a Fire i400 camera (Unibrain, San Ramon, CA, USA) mounted on Nikon SMZ800 stereomicroscope (Gx9.8). Twenty second-long live videos were digitized at 30 frames.s^−1^ (total of 600 collected frames) using PhotoBooth and Nikon software and calibrated using a stage micrometer. Briefly, larvae were transiently immobilized in 2% methyl cellulose and oriented to optimize the viewing angle of the beating ventricle through the transparent body wall. Video clips of the beating heart were recorded. The 20 s videos, which would have captured up to 60-70 heart beats at control heart rate levels, were then analyzed, frame-by-frame with Image J software to calculate heart rate, stroke volume and cardiac output. Heart rate was assessed as the number of heartbeats recorded in three sequential 10 s periods of video, which was then averaged and multiplied by six to generate mean heart rate in beats/min. Similarly, the beating heart was assessed for end-diastolic and end-systolic cross-sectional areas of the beating ventricle in the sagittal plane of view. Using dimensional analysis that assumes the heart to be a prolate spheroid (Bagatto and Burggren, 2006; Perrichon et al., 2017), the end-diastolic and end-systolic volumes were calculated and stroke volume determined by subtracting the smaller end-systolic volume from the larger end-diastolic volume (and assuming heart tissue is non-compressible). Cardiac output, in turn, was calculated as the product of heart rate and stroke volume. Heart rate, stroke volume, and cardiac output were determined immediately prior to severe hypoxia exposure and cardiac arrest, and at 2 h, 24 h and 72 h following the resumption of heartbeat.

### Immunohistochemistry

To provide a qualitative assessment of whether the heart tissues of zebrafish larvae exhibited signs of apoptosis or cell damage after induction of cardiac arrest, immunohistochemical staining was performed on ventricular tissues taken from three control larvae and from five experiencing cardiac damage from severe hypoxia. A combination of cardiomyocyte- and general tissue-specific stains were used (Amin et al., 2011). Briefly, larval zebrafish were sampled 2 h after the heart stopped (cardiac arrest) in severe hypoxia (4 kPa). Larvae were euthanized with MS-222 solution (300 mg/l buffered to pH 7.4 with 1 M HCO_3_) and immediately fixed in Z-fix (zinc-buffered formalin – Anatech Ltd.) for 48 h. After fixation larvae were dehydrated, cleared and embedded in Paraplast Extra, then sliced into 4 µm sections with a rotary microtome (Leica RM2245). Sections were mounted on charged slides and dried prior to treatment.

To confirm apoptosis and identify cell proliferation, whole zebrafish larvae were sectioned along the sagittal plane to produce between 5-10 serial sections per slide. Immunohistochemistry (IHC) methods and protocols were set up on the Ventana Discovery Ultra autostainer, which gives high sensitivity, specificity and reproducible results. Duplex immunohistochemistry for detection of Cardiac Troponin T (cTropT) and Cleaved Caspase 3 (CC3) was carried out according to manufacturer's recommendations and all reagents except antibodies were Ventana products (Roche, Basel, Switzerland). Antigen retrieval was done in Ventana using Cell Conditioner1 (CC1; catalog number 6414575001) for 40 min at 95°C. Anti-CC3 primary antibody was added for 1 h at 37°C (dilution 1:100, #9664, Cell Signaling Technology, Boston, MA, USA) followed by secondary DISCOVERY OmniMap anti-Rb HRP RUO (catalog number 5269679001) and Purple chromogenic detection (DISCOVERY Purple Kit RUO (catalog number 7053983001). After antibody denaturation using ULTRA Cell Conditioning (CC2) (catalog number 5424542001) for 8 min at 100°C, cTropT was added for 1 h at 37°C (dilution 1:200, MA5-12960, Invitrogen/ThermoFisher Scientific, Waltham, MA, USA) followed by Anti-Mouse-NP (Nitropyrazole)(catalog number 7425317001), anti-NP (catalog number 7425317001) and Discovery Yellow chromogenic detection (catalog number 7698445001).

To ensure that stained material was specifically cardiac muscle that was showing damage, additional staining on three 7 dpf larvae was conducted to co-localize biomarkers for cell damage in cardiac muscle cells. As a marker for apoptosis, slides with heart tissue from the ventral wall of the ventricle were treated with conjugated monoclonal antibodies for Caspase-3 [Caspase-3 (CJPP364-1-18), Santa Cruz Biotechnology, sc-56052 AF594]. Conjugated monoclonal antibodies (mAb) were also also used against bovine cardiac muscle troponin T [Troponin T-C (CT3), Santa Cruz Biotechnology, sc-20025 AF488] and Caspase-3. Co-localization of Caspase-3 with troponin T indicates that cardiomyocytes were experiencing apoptosis and the associated degradation. Antigen retrieval was carried out at low pH in citrate buffer with three 2 min bursts of high heat in a microwave on high, followed by a 2 min cooldown at room temperature between heating cycles. After the third heat cycle, slides were allowed to cool to room temperature before treatments. After cooldown, slides were again rinsed three times in PBS. Slides were then treated with blocking buffer (2% fetal bovine serum and 1% horse serum in PBS) for 30 min. Following blocking, the CT3 antibody was diluted in blocking buffer and applied at 1:5000 dilution. Slides were incubated with CT3 antibody overnight at 4°C. The following morning, slides were rinsed in blocking buffer, then were treated with a 1:10,000 dilution of Caspase-3 for 1 h at room temperature. Slides were rinsed as before, then treated with Phalloidin 350 (Vector Laboratories) as a counter stain, per manufacturer's instructions.

Note that the IHC performed in this study was qualitative rather than quantitative in nature, and images were produced for illustrative purposes rather than to quantify the overall extent of cell apoptosis or proliferation.

### Statistics

Individual one-way analysis of variance (ANOVA) tests were used to analyze for effects of larval age on time to loss of mobility, time to cardiac arrest, and time for resumption of heart rate. Each of these variables was designated as a single factor in the analysis. Survival following cardiac arrest was assessed with a Fischer's Exact Test. Data for effects of cardiac arrest on heart rate, stroke volume and cardiac output were analyzed separately with a two-way ANOVA, with treatment (control, hypoxic-exposure) and time as the two factors. *Post-hoc* comparisons were performed with a Holm-Sidak procedure for pair-wise comparisons. All data are expressed as mean±s.e.m., and significance level was considered when *P*<0.05 for all tests. Statistical testing and plotting were performed with SigmaStat 15 (Systat Software Inc.).

### Compliance with ethical regulations

All experiments were approved and performed in strict accordance with the Institutional Animal Care and Use Committee (IACUC-Protocol #15003) at the University of North Texas. Additionally, all procedures conform to the guidelines from Directive 2010/63/EU of the European Parliament on the protection of animals used for scientific purposes or the NIH Guide for the Care and Use of Laboratory Animals.
